# The antimicrobial peptide Esc(1-21)-1c increases susceptibility of *Pseudomonas aeruginosa* to conventional antibiotics by decreasing the expression of the MexAB-OprM efflux pump

**DOI:** 10.3389/fchem.2023.1271153

**Published:** 2023-10-24

**Authors:** Carolina Canè, Bruno Casciaro, Angela Di Somma, Maria Rosa Loffredo, Elena Puglisi, Gennaro Battaglia, Marta Mellini, Floriana Cappiello, Giordano Rampioni, Livia Leoni, Angela Amoresano, Angela Duilio, Maria Luisa Mangoni

**Affiliations:** ^1^ Department of Chemical Sciences, University of Naples “Federico II”, Naples, Italy; ^2^ Laboratory Affiliated to Pasteur Italia-Fondazione Cenci Bolognetti, Department of Biochemical Sciences, Sapienza University of Rome, Rome, Italy; ^3^ CEINGE Biotecnologie Avanzate, Naples, Italy; ^4^ Department of Science, University “Roma Tre”, Rome, Italy; ^5^ IRCCS Fondazione Santa Lucia, Rome, Italy; ^6^ National Institute of Biostructure and Biosystems (INBB), Rome, Italy

**Keywords:** *Pseudomonas aeruginosa*, antimicrobial peptide, antibiotics, tetracycline, efflux pumps, proteomic

## Abstract

**Introduction:** The increase in bacterial strains resistant to conventional antibiotics is an alarming problem for human health and could lead to pandemics in the future. Among bacterial pathogens responsible for a large variety of severe infections there is *Pseudomonas aeruginosa*. Therefore, there is an urgent need for new molecules with antimicrobial activity or that can act as adjuvants of antibiotics already in use. In this scenario, antimicrobial peptides (AMPs) hold great promise. Recently, we characterized a frog-skin AMP derived from esculentin-1a, namely Esc(1-21)-1c, endowed with antipseudomonal activity without being cytotoxic to human cells.

**Methods:** The combinatorial effect of the peptide and antibiotics was investigated through the checkerboard assay, differential proteomic and transcriptional analysis.

**Results:** Here, we found that Esc(1-21)-1c can synergistically inhibit the growth of *P. aeruginosa* cells with three different antibiotics, including tetracycline. We therefore investigated the underlying mechanism implemented by the peptide using a differential proteomic approach. The data revealed a significant decrease in the production of three proteins belonging to the MexAB-OprM efflux pump upon treatment with sub-inhibitory concentration of Esc(1-21)-1c. Down-regulation of these proteins was confirmed by transcriptional analysis and direct measurement of their relative levels in bacterial cells by tandem mass spectrometry analysis in multiple reaction monitoring scan mode.

**Conclusion:** These evidences suggest that treatment with Esc(1-21)-1c in combination with antibiotics would increase the intracellular drug content making bacteria more susceptible to the antibiotic. Overall, these results highlight the importance of characterizing new molecules able to synergize with conventional antibiotics, paving the way for the development of alternative therapeutic strategies based on AMP/antibiotic formulations to counteract the emergence of resistant bacterial strains and increase the use of “old” antibiotics in medical practice.

## 1 Introduction

Nowadays, antimicrobial resistance is one of the greatest challenges to human health worldwide. Misuse and overuse of antibiotics has led to the spread of multidrug-resistant strains that have acquired new resistance mechanisms (Mancuso et al., 2021). As a result, antibiotics and other antimicrobial drugs have become ineffective, threatening our ability to treat common infections. Over the past decade, the number of microbes resistant to several classes of available antimicrobial agents, and therefore defined as superbugs, has significantly increased, making many infections difficult or impossible to treat (Abushaheen et al., 2020). The term “ESKAPEE” includes seven bacterial pathogens responsible for a large number of nosocomial infections and capable of “escaping” the biocidal action of antibiotics (Rice, 2008; Aloke and Achilonu, 2023): *Enterococcus faecium, Staphylococcus aureus, Klebsiella pneumoniae, Acinetobacter baumannii, Pseudomonas aeruginosa*, *Enterobacter* spp., and *Escherichia coli* (Rice, 2008; Ruekit et al., 2022). Overall, the need of saving humankind from further pandemics is highly pressing.

Alternative therapies currently in clinical trials or routine use include the use of antibiotics in combination with other compounds able to potentiate their effects. Among these compounds, antimicrobial peptides (AMPs) are very promising (Pizzolato-Cezar et al., 2019; Sheard et al., 2019; Sajid et al., 2022). AMPs are cationic molecules containing 10 to 50 amino acids and represent important components of innate immunity in almost all living organisms. They exhibit a broad spectrum of activity against a large variety of microorganisms with a membrane-perturbing mechanism of bactericidal activity (Hancock et al., 2021; Kim et al., 2023).

Recently, a short peptide corresponding to the first 20 residues of the frog skin AMP esculentin-1a, and carrying a glycine amide at its C-terminus, namely, Esc(1-21), as well as its diastereomer Esc(1-21)-1c containing two d-amino acids (i.e., d-Leu^14^ and d-Ser^17^) were identified and characterized. Both Esc(1-21) and Esc(1-21)-1c peptides displayed significant antipseudomonal activity at concentrations ranging from 1 to 16 µM through perturbation of the cytoplasmic membrane of either planktonic or sessile bacterial forms (Loffredo et al., 2017). The change of l-to d-amino acids was proposed to confer the peptide 1) greater resistance to proteolytic degradation; and 2) lower cytotoxicity against eukaryotic cells (Di Grazia et al., 2015). The capability of Esc(1-21)-1c to inhibit *P. aeruginosa* biofilm formation at concentrations lower than those killing the microbe was also demonstrated. This antibiofilm activity is dependent on the ability of Esc(1-21)-1c to hamper bacterial motility and to decrease the expression of virulence genes (Casciaro et al., 2019). In addition, when tested at sub-inhibitory concentrations, Esc(1-21)-1c was able to synergize with the conventional antibiotic aztreonam in inhibiting growth and in killing *P. aeruginosa* cells (Casciaro et al., 2018). Despite combined therapy can lead to disadvantages such as increased expense and risk of adverse effect (Traugott et al., 2011), incompatible pharmacokinetics, antagonism and superinfections (Rybak and McGrath, 1996; Bassetti et al., 2008), the use of AMP and antibiotics (leading to synergistic effect), allows a therapeutic outcome at lower doses of each single drug. Compared to single drug therapy, the combination of drugs could reduce the overall toxicity of treatment and also the likelihood of resistance development (Fischbach, 2011). On this ground, studies addressing the effect of sub-inhibitory concentrations of Esc(1-21)-1c and/or other AMPs on the protein expression profile of ESKAPEE-listed microbial pathogens are essential to expand our knowledge on 1) the mechanism(s) of antimicrobial activity of AMPs at doses that do not affect the bacterial viability, and 2) the plausible mechanism underlying their synergism in combination with conventional antibiotics. Both issues have not been widely explored so far. In fact, despite valuable examples of combinatory therapy based on AMP/antibiotic have been provided in the last years (Santos et al., 2022), studies addressing the molecular mechanism underlying the capability of AMPs to boost the effect of antibiotics are very limited.

Here, we investigated the combinatorial effect of Esc(1-21)-1c with a panel of antibiotics belonging to various drug classes (i.e., ceftazidime, tobramycin, tetracycline, erythromycin, and chloramphenicol) on the reference strain *P. aeruginosa* PAO1. Moreover, starting from the *in vitro* results showing a synergistic activity in inhibiting bacterial growth, we explored the effect of sub-MIC of Esc(1-21)-1c on *P. aeruginosa* expression profile by differential proteomic analysis. Our data indicated a significant decrease in the production of the MexA, MexB and OprM proteins belonging to the efflux pump responsible for the extrusion of antibiotics from bacterial cells (Lopez et al., 2017). These results were also confirmed by transcriptional analysis showing a decreased expression of the corresponding genes and by quantitative evaluation of both the antibiotic and the selected proteins inside bacterial cells by tandem mass spectrometry analysis in multiple reaction monitoring (MRM) scan mode.

These data provide the first evidence of a mechanism responsible for the higher susceptibility of *P. aeruginosa* to some antibiotics upon exposure to Esc(1-21)-1c, opening the way for the development of novel AMP/antibiotics formulations. This will accelerate the traditional process of drug development by potentiating “old” antibiotics already known to be safe and effective in humans.

## 2 Results

### 2.1 Combination of Esc(1-21)-1c with antibiotics

The combinatorial effect of Esc(1-21)-1c peptide with different antibiotics belonging to various classes was evaluated by the checkerboard titration assay on *P. aeruginosa* PAO1 to determine the fractional inhibitory concentration index (FICI, Supplementary Equations S1–S5). The antibiotic(s) and the peptide were added either individually or in combination, in a serial-two-fold dilution, to the wells of a 96-multiwell plate containing bacterial cells. Table 1 reports the effect of Esc(1-21)-1c in combination with different antibiotics on the MIC of both compounds in comparison to the MIC of each compound when used individually. The FICI was interpreted as follows: FICI ≤0.5, synergy; 0.5 < FICI ≤1, additivity; 1 < FICI ≤2, no interaction; FICI >2, antagonism (Pollini et al., 2017; Casciaro et al., 2018).

**TABLE 1:** Effect of Esc(1-21)-1c and various antibiotics on *P. aeruginosa* PAO1, either used individually or in combination.

Molecules	MIC alone µg/mL [µM]	MIC in combination µg/mL [µM]	FICI
Esc(1-21)-1c	6.8 [3.12]	6.8 [3.12]	2
Tobramycin	1 [2.14]	1 [2.14]	
Esc(1-21)-1c	6.8 [3.12]	3.4 [1.56]	0.625
Ceftazidime	8 [14.65]	1 [1.82]	
Esc(1-21)-1c	6.8 [3.12]	1.7 [0.78]	0.375
Erythromycin	256 [348.8]	32 [43.6]	
Esc(1-21)-1c	6.8 [3.12]	0.85 [0.39]	0.25
Chloramphenicol	32 [99]	4 [12.4]	
Esc(1-21)-1c	6.8 [3.12]	1.7 [0.78]	0.375
Tetracycline	8 [18]	1 [2.25]	

**FIGURE 1:**
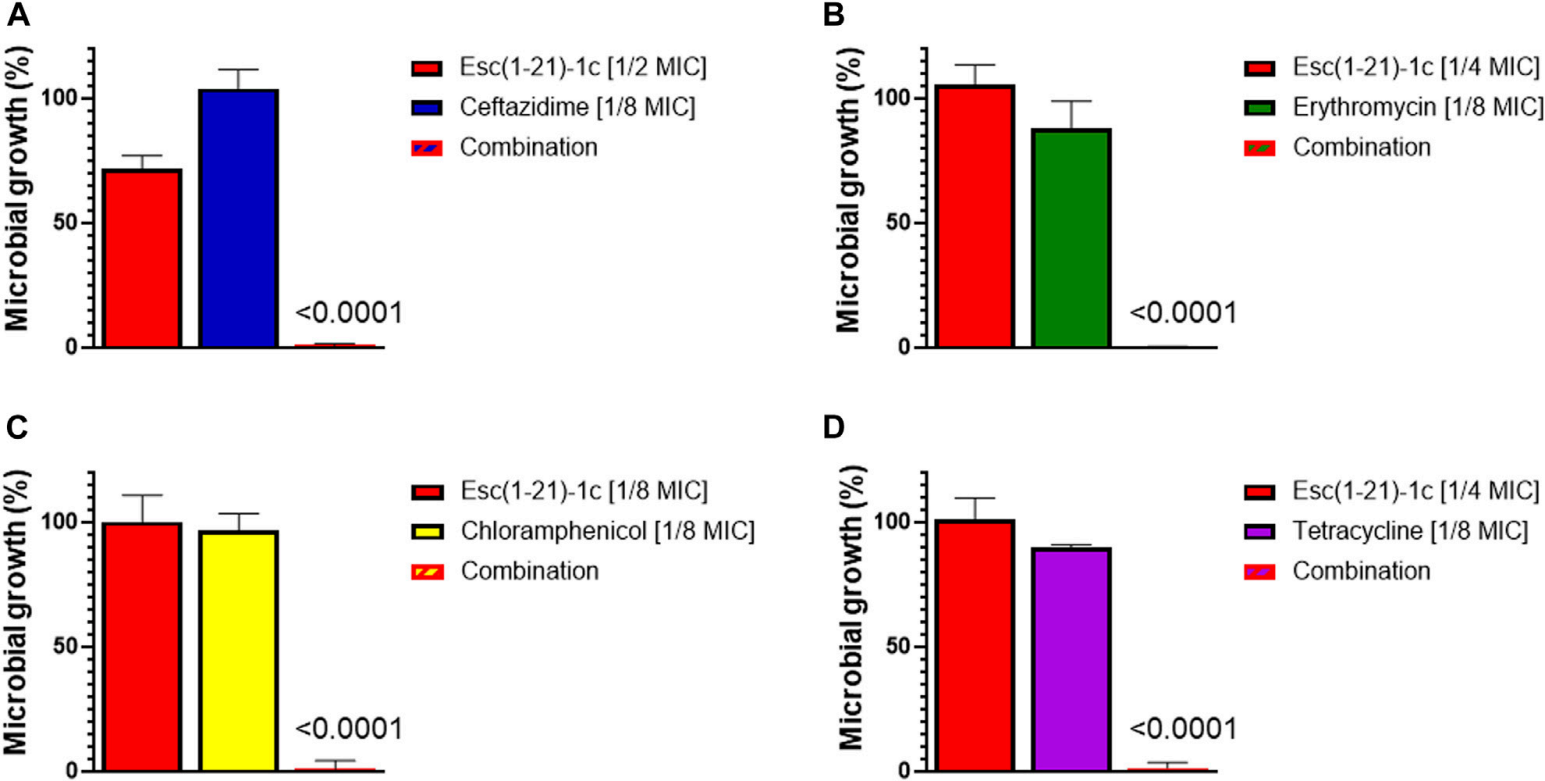
Growth of *P. aeruginosa* PAO1 (spectrophotometrically measured as optical density) after 16 h treatment with antibiotics or Esc(1-21)-1c, either alone or in combination. Bacterial growth was expressed as percentage compared to that of untreated control cells (100%). **(A)** ceftazidime; **(B)** erythromycin; **(C)** chloramphenicol; **(D)** tetracycline. The data are the mean ± standard deviation (SD) of three independent experiments. The level of statistical significance (*p* values) were calculated by performing one-way analysis of variance (ANOVA) and reported in the figure; ns, not statistically significant (*p* > 0.5).

As indicated in Table 1, Esc(1-21)-1c displayed a clear synergistic effect when combined with erythromycin (FICI = 0.375), chloramphenicol (FICI = 0.25), and tetracycline (FICI = 0.375). Moreover, an additive effect (FICI = 0.625) was obtained for the mixture of the peptide with ceftazidime, whereas Esc(1-21)-1c did not show any synergistic or additive effect when mixed with tobramycin (FICI = 2). The growth of *P. aeruginosa* PAO1 treated with ceftazidime, erythromycin, chloramphenicol, and tetracycline in combination with the peptide (at its best concentration giving a synergistic/additive effect, as indicated in Table 1) are reported in Figures 1A–D. Bacterial cells were clearly able to grow in the presence of either the antibiotic or the peptide alone whereas no growth was observed when *P. aeruginosa* was treated with each single antibiotic in combination with Esc(1-21)-1c. Notably, when the combination of Esc(1-21)-1c with tetracycline was tested against a tetracycline-resistant clinical isolate of *P. aeruginosa* the synergistic activity was preserved (FICI = 0.375).

### 2.2 Differential proteomic analysis

These results prompted us to investigate the global effect elicited by the peptide on *P. aeruginosa* proteome following treatment with Esc(1-21)-1c at sub-MIC concentration. To this end, a differential proteomic experiment was designed according to the label-free procedure to compare the protein expression of *P. aeruginosa* PAO1 before and after Esc(1-21)-1c treatment. *P. aeruginosa* PAO1 cells were incubated with 60 μg/mL (25 µM) of Esc(1-21)-1c (a peptide concentration that does not significantly affect the bacterial growth, Supplementary Figure S1–S5) for 3 hours. A significantly higher number of bacterial cells was necessary for this experiment compared to the checkerboard assay. Cells were then lysed, and the protein content was purified by solid-phase extraction and hydrolysed with trypsin. Peptide mixtures for each condition were analysed in duplicate by nano Liquid Chromatography/Tandem Mass Spectrometry (LC-MS/MS). Raw data were used for protein identification and quantification by using MaxQuant search engine and the specific *P. aeruginosa* protein sequence database. Statistical analysis returned a total of 108 significantly differentially expressed proteins (*p* < 0.01). By considering a log_2_ fold change (FC) cutoff between −0.5 and 0.5, 57 proteins resulted upregulated and 51 downregulated in the Esc(1-21)-1c treated samples in comparison to the control.

**FIGURE 2:**
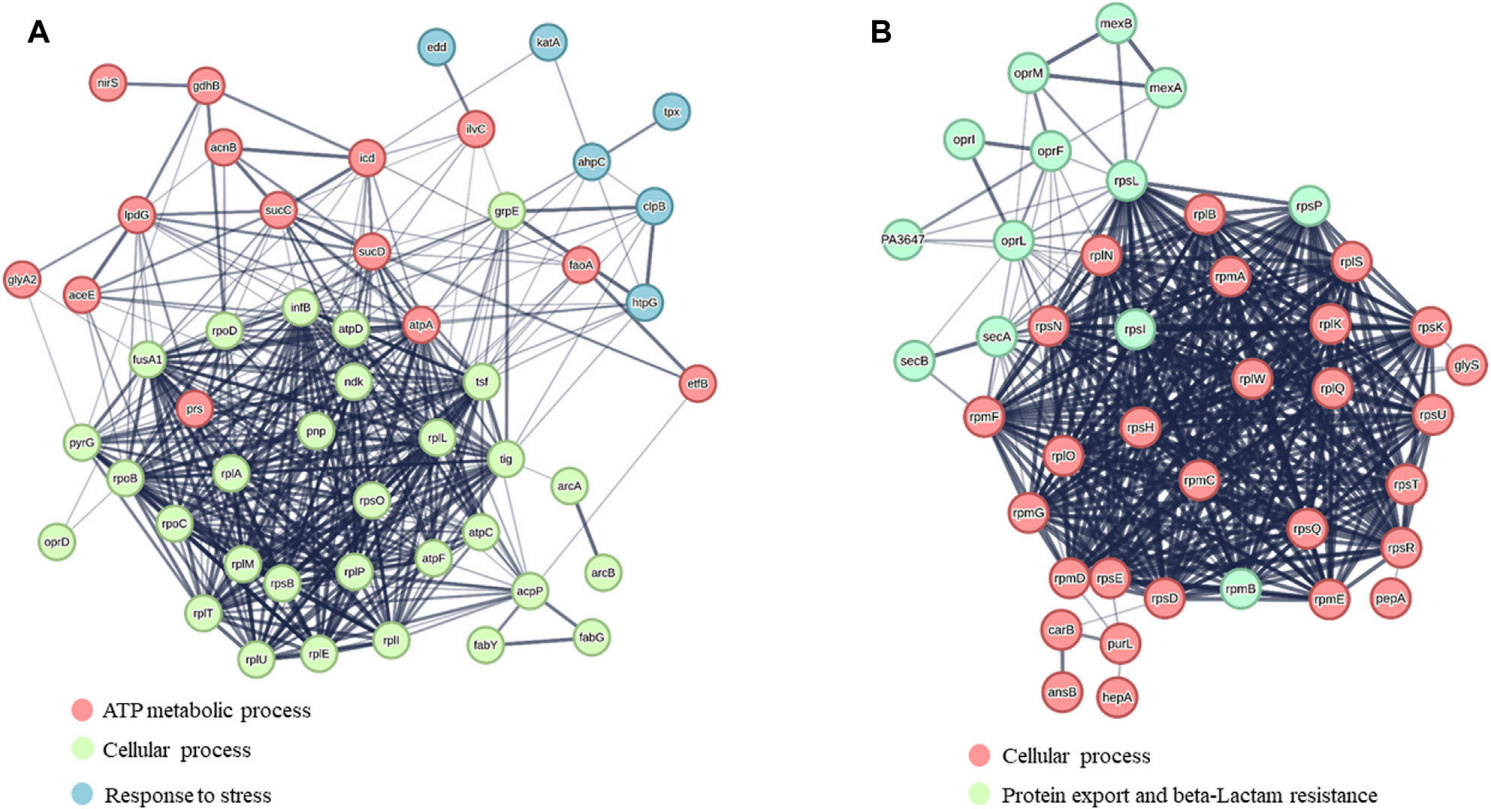
STRING analysis of **(A)** upregulated proteins and **(B)** downregulated proteins. Protein clusters were formed based on the major biological processes in which proteins are involved.

Supplementary Table S1, S2 report the protein name, the corresponding SwissProt code, gene name and FC calculated for each identified protein. Bioinformatic analysis was performed using the software STRING and the up- and downregulated proteins were grouped according to the interactions they are engaged (Figure 2).

Besides several accessory proteins, ribosomal proteins, metabolic enzymes, chaperones, etc., the differential proteomics experiment revealed a number of proteins whose levels were significantly affected by Esc(1-21)-1c treatment. Among them, we focused on the proteins involved in the influx/efflux mechanism of antibiotic molecules, to find an explanation for the biological evidence shown in Table 1. They are listed in Table 2. Among the downregulated proteins, the membrane proteins MexA, MexB and OprM emerged. Together, they form a well-known tripartite efflux pump of *P. aeruginosa* and are encoded by the *mexA-mexB-oprM* operon, which is involved in multidrug resistance (Lorusso et al., 2022). Downregulation of this efflux pump by Esc(1-21)-1c treatment could then contribute significantly to the reduction of intrinsic antibiotic resistance in *P. aeruginosa*. Moreover, SecA, SecB, OprF and OprI proteins involved in the secretion of proteins, antibiotics, and metabolites were also downregulated, possibly decreasing the capability of *P. aeruginosa* to extrude antimicrobial drugs. Note that decreased production of essential secretion system and ribosomal proteins is likely responsible for the slower bacterial growth (than untreated samples) that can be appreciated only after a longer time treatment with the peptide (16 h, Supplementary Figure S1) compared to the 3 h (the time used for proteomic studies) during which bacterial growth was not significantly affected.

**TABLE 2:** List of proteins up- or downregulates involved in the influx/efflux mechanism of antibiotic molecules.

SwissProt code	Protein name	Gene	Effect	FC
P32722	Porin D	*oprD*	+	1.45
P52477	Multidrug resistance protein MexA	*mexA*	-	0.78
Q9HU56	Protein-export protein SecB	*secB*	-	0.72
Q9LCT3	Protein translocase subunit SecA	*secA*	-	0.56
P13794	Outer membrane porin F	*oprF*	-	0.40
Q51487	Outer membrane protein OprM	*oprM*	-	0.53
P52002	Multidrug resistance protein MexB	*mexB*	-	0.29
P11221	Outer membrane lipoprotein I	*oprI*	-	0.22

+, upregulated; -, downregulated.

Interestingly, differential proteomic investigation showed an increase in OprD, the major porin of *P. aeruginosa* involved in antibiotic uptake. According to the literature, the absence of OprD in *P. aeruginosa* increases resistance to antibiotics (Li et al., 2012).

### 2.3 Transcriptional analysis

Based on the proteomic results, we then explored whether the effect of the peptide was exerted either by modulating gene transcription or by other post-transcriptional mechanisms. To this end, mRNA levels of the selected genes corresponding to the specific altered proteins were examined in Esc(1-21)-1c-treated and untreated *P. aeruginosa* PAO1 cultures by means of RT-qPCR analysis.

**FIGURE 3:**
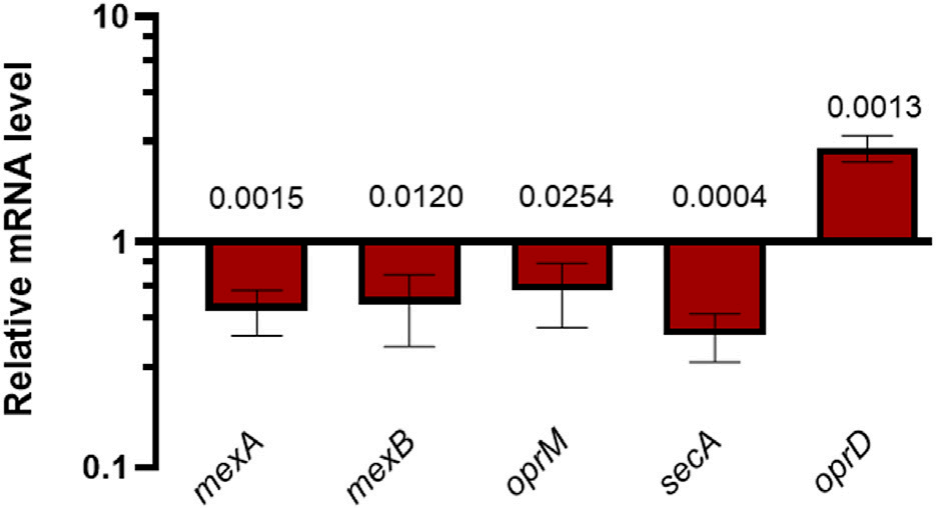
RT-qPCR measurements of specific mRNAs in *P. aeruginosa* PAO1 cultures treated with 25 µM Esc(1-21)-1c relative to untreated *P. aeruginosa* PAO1 cultures. The average of three independent experiments is reported with SD. Significance with respect to the untreated control samples was calculated by performing the *t*-test analysis and reported in the figure; ns, not statistically significant.

Figure 3 clearly shows that the AMP treatment led to a significant downregulation of the expression of the *mexA*, *mexB* and *oprM* genes belonging to the *mexAB*-*oprM* operon, as well as the *secA* gene. Moreover, the expression of *oprD* was upregulated in Esc(1-21)-1c-treated cultures compared with untreated controls. These data are consistent with proteomic results and support the hypothesis that Esc(1-21)-1c treatment increases antibiotic uptake *via oprD* overexpression, and limits antibiotic efflux *via* decreased expression of the *mexA*, *mexB*, *oprM* and *secA* genes.

### 2.4 Quantitative analyses of tetracycline within *P. aeruginosa* cells

The Esc(1-21)-1c-mediated reduction in MexAB-OprM efflux pump expression may explain the ability of the peptide to potentiate the effects of several antibiotics, including tetracycline, by retaining their intracellular content. To date, tetracycline is used in clinical practise, confirming its potential as an antibiotic (LaPlante et al., 2022). Therefore, we addressed the quantitative analysis of tetracycline taken up by *P. aeruginosa* cells in the presence and in the absence of Esc(1-21)-1c. For this purpose, a tandem mass spectrometry method based on MRM scan mode was developed. First, tetracycline standard solutions were analysed at various concentrations using LC-MS/MS in MRM conditions to determine the optimal instrument settings and define the best parent ion-fragment ion mass transitions. These analyses led to the construction of calibration curves elaborated by linear functions showing a coefficient of determination (R2) greater than 0.99.

**FIGURE 4:**
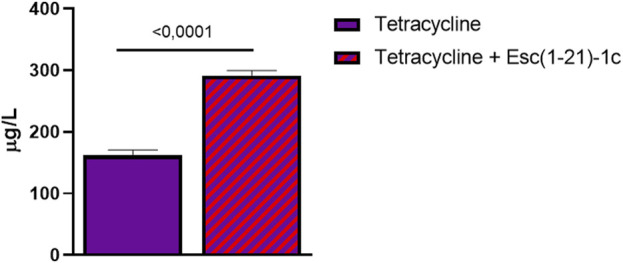
Relative amounts of tetracycline detected within *P. aeruginosa* cells in samples treated with the antibiotic alone (violet bar) and with a combination of antibiotic plus Esc(1-21)-1c (violet + red bar). Data represent the mean ± SD from three independent experiments; the level of statistical significance among groups was calculated by performing the *t*-test analysis and indicated in the figure.

Metabolites were then extracted from *P. aeruginosa* cells treated with either tetracycline alone or with the combination of Esc(1-21)-1c plus tetracycline for 3 h, and the amount of tetracycline inside bacterial cells was evaluated in treated samples by means of the developed MRM conditions. Figure 4 shows the quantitative analysis of tetracycline extracted from the samples. Results showed that tetracycline levels in the bacterial cells treated with the mixture of Esc(1-21)-1c plus tetracycline were about double compared to the level found in samples treated with tetracycline alone (∼297 vs. ∼162 μg/L, respectively). This result supports the hypothesis that the administration of Esc(1-21)-1c in combination with certain antibiotics increases their uptake and/or decreases their efflux.

### 2.4 Relative quantification of MexA, MexB, and OprM from *P. aeruginosa* upon treatment with tetracycline alone or in combination with the peptide

**FIGURE 5:**
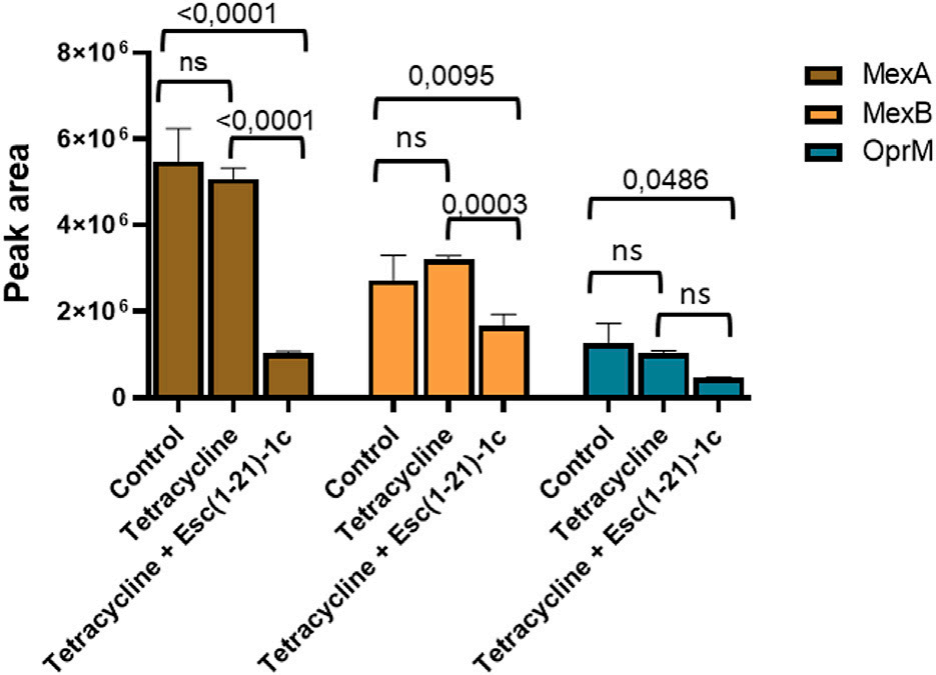
Quantitative measurements of MexA (brown bars), MexB (orange bars) and OprM (cyan bars) by LC-MS/MS in MRM mode in *P. aeruginosa* cells treated with either the antibiotic alone or a combination of antibiotic plus Esc(1-21)-1c. Data represents the mean ± SD; the level of statistical significance among groups was calculated by performing the two-way analysis of variance (ANOVA) and indicated in the figure.

As mentioned above, proteomic and transcriptional analyses proved the downregulation of the MexA, MexB, and OprM proteins and the corresponding genes, respectively. This was also supported by the evaluation of the amount of these proteins in bacterial cells following incubation with tetracycline alone or in combination with Esc(1-21)-1c, by MRM tandem mass spectrometry. The *in silico* analysis carried out using Skyline software (Supplementary Table S3), allowed us to identify specific precursor ion-product ion transitions to select a unique peptide for each target protein. The specific peptides used to monitor the three target proteins are reported in Materials and Methods. Proteins were extracted from *P. aeruginosa* PAO1 cultures treated with tetracycline alone or in combination with Esc(1-21)-1c, and from untreated cultures, as control. Protein extracts were digested with trypsin and analysed in triplicate by LC-MS/MS in MRM scan mode monitoring the ion current associated with the mass transitions for the 3 selected peptides (see Materials and Methods). The average peak areas from the different replicates were recorded for each peptide from MexA, MexB, and OprM. Quantitative evaluation of the MRM/MS data demonstrated a significant reduction in the amount of the monitored proteins in tetracycline plus Esc(1-21)-1c-treated cultures (Figure 5), confirming previous data on the decreased expression of the *mexAB-oprM* genes. Conversely, tetracycline alone did not affect the level of the analysed proteins.

## 3 Discussion

The development of adjuvants targeting antibiotic resistance mechanisms to re-evaluate the efficacy of conventional antibacterial agents is one of the therapeutic strategies to address the current global health problem related to the widespread of multi-drug resistant (MDR) strains, including *P. aeruginosa*, one of the most important causative agents of respiratory infections, especially in cystic fibrosis patients (Wright, 2016; Domalaon et al., 2018). Previous studies have shown that combining antibiotics with some AMPs can have a synergistic effect, increasing antibiotic activity while preventing bacterial resistance (Maisetta et al., 2009; Gupta et al., 2015). In the Gram-negative bacterium *P. aeruginosa*, one of the mechanisms associated with MDR is the extrusion of antibiotics from bacterial cells, generally mediated by efflux pumps of the Resistance-Nodulation-Division (RND) family (Nikaido, 1998b; Nikaido, 1998a). These consist of a tripartite complex formed by a transmembrane protein in the cytoplasmic membrane, a periplasmic protein, and a porin associated to the outer membrane (Lorusso et al., 2022).

**FIGURE 6:**
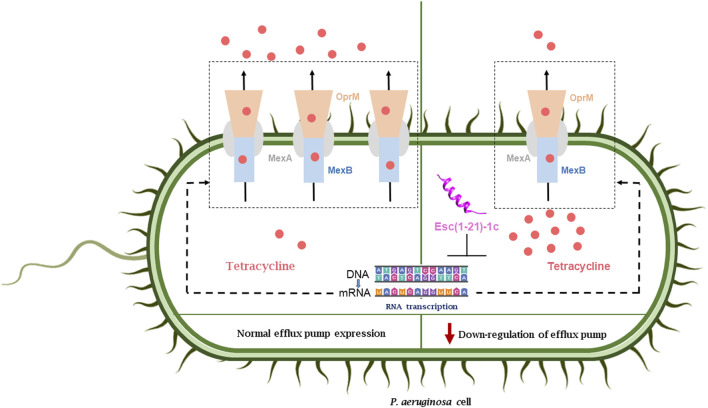
Effect of peptide treatment on the expression of the MexAB-OprM efflux pump and the consequent accumulation of antibiotics inside the bacterial cell (right side) with respect the normal condition (left side).

The present study has demonstrated that the combination of Esc(1-21)-1c with erythromycin, chloramphenicol, or tetracycline had a synergistic effect in inhibiting the growth of *P. aeruginosa* PAO1, whereas only an additive effect was recorded for the peptide in combination with ceftazidime. In comparison, an indifferent effect resulted when Esc(1-21)-1c was tested in the presence of tobramycin. By means of differential proteomic experiments, we discovered an alteration in the mechanisms for drug uptake/efflux in *P. aeruginosa* cells, following incubation with Esc(1-21)-1c in its sub-MIC. Specifically, the expression level of MexAB-OprM efflux pump components was decreased, while the expression of OprD protein, which is involved in outer membrane permeability (Li et al., 2012), was significantly increased. These data were confirmed by transcriptomic analysis of the corresponding genes, and further supported by direct measurements of the amount of the specific proteins, using LC-MS/MS procedures in the MRM scan mode. A schematic representation of the effect of Esc(1-21)-1c on the expression of MexAB-OprM is reported in Figure 6.

The MexAB-OprM system plays a central role in the multidrug resistance mechanism of *P. aeruginosa* by extruding a large variety of antimicrobials encompassing β-lactams, macrolides, chloramphenicol, tetracyclines, streptogramin, quinolones (including ciprofloxacin and nalidixic acid) (Poole, 2001; Moreira et al., 2004; Blair et al., 2014; Ma et al., 2021; Nishino et al., 2021). When the drug concentration near the pump increases, the MexB protein undergoes conformational changes to eject the active drug across the periplasm and outer membrane through the tunnel formed by the other two proteins of the pump, i.e., MexA and OprM (Tsutsumi et al., 2019). This complex is highly conserved and frequently overexpressed in clinical MDR isolates of *P. aeruginosa*, corroborating its role in the intrinsic resistance of these bacteria to currently used antibiotics (Jeannot et al., 2008; Stickland et al., 2010). Overproduction of the MexAB-OprM efflux system due to mutations in the *nalB* repressor gene makes *P. aeruginosa* more resistant to several antibacterial agents (Jo et al., 2003). On the contrary, genetic deletion of this efflux pump results in impressive reduction in MICs of antibiotics that are not considered to have clinically significant antipseudomonal activity (Nikaido, 1998b; Nikaido, 1998a; Li et al., 2000).

Impairment of the efflux activity of the MexAB-OprM pump, and enhanced uptake activity *via* the OprD porin would increase drug content in intact cells, and make the bacteria more susceptible to the antibiotic. This was confirmed by direct measurement of tetracycline content in *P. aeruginosa* cells exposed to Esc(1-21)-1c by LC-MS/MS MRM analyses, pointing out to a significant higher tetracycline level in peptide-treated samples compared with the controls. The accumulation of tetracycline and norfloxacin was also reported to be higher in *mexA* and *oprM* null mutants, which in turn displayed a greater susceptibility to a wide variety of beta-lactam antibiotics (Li et al., 1995). Notably, it cannot be excluded that the effect of an increased tetracycline level inside the cells is also due to the membrane permeabilization caused by the peptide. To date, the drug impermeable bacterial membrane (especially in Gram-negative bacteria) limits antibiotic access. For this reason, improvement of the intracellular uptake of drugs has a great demand and the effect of Esc(1-21)-1c may contribute to achieve a higher intracellular level of antibiotics.

Interestingly, our experiments revealed no synergistic effect when Esc(1-21)-1c was combined with tobramycin. This is reasonable, considering that resistance to aminoglycosides in *P. aeruginosa* mainly results from the activity of the modifying enzyme aminoglycoside nucleotidyl-transferase rather than active efflux (Pang et al., 2019).

Remarkably, our results well agree with those reported by Shang and colleagues for the Trp-containing peptides L11W and L12W, which significantly inhibited the expression of *oprM* and *mexA* genes in *P. aeruginosa* cells, while raising the transcription of the *oprD* gene as well as the intracellular content of antibiotics such as ceftazidime (Shang et al., 2021).

We did not record a synergistic effect of Esc(1-21)-1c with ceftazidime, but rather an additive effect. Beta-lactams act on cell wall synthesis; they do not need to enter the cytoplasm to exert their function. Considering the mechanism of action of Esc(1-21)-1c as an adjuvant, it makes sense that it has not significant effect on the MIC of this class of compounds.

Finally, the downregulation of SecA, SecB, OprF and OprI proteins supports the hypothesis that the presence of Esc(1-21)-1c may impair the ability of *P. aeruginosa* to export antibiotics, which has a number of negative effects on the survival of the bacterium.

Further investigations are needed to define the specific role of Esc(1-21)-1c in affecting the expression of efflux pump components, although negative transcriptional control can be supposed based on the transcriptional data. The MexAB-OprM pump is controlled by both local and global regulators (Aguilar-Rodea et al., 2022). The reduced expression of the pump could be due to the interaction of the peptide with some molecules involved in the regulatory mechanisms of the MexAB-OprM efflux system (Aguilar-Rodea et al., 2022).

Nowadays, the development of efflux pump inhibitors (EPIs) constitutes an important area of drug discovery, and great perspectives can be foreseen for their use to inhibit or limit the emergence of multidrug resistance bacteria (Van Bambeke et al., 2003; Stavri et al., 2007).

## 4 Materials and Methods

### 4.1 Chemicals and bacterial strain

The peptide Esc(1-21)-1c was synthetically produced by Biomatik (Wilmington, NC, United States). Synthesis was by stepwise solid-phase technique, using a standard F-moc protocol. Purification (to 95%) was performed via reverse-phase high-performance liquid chromatography (RP-HPLC), and molecular mass was verified by mass spectrometry. The bacterial strain used was the reference *P. aeruginosa* PAO1 (ATCC 15692) and a tetracycline-resistant clinical isolate named 24717(1), kindly provided by Prof. Giammarco Raponi from the strain collection of Policlinico Umberto I. The antibiotics were purchased from Sigma-Aldrich (Milan, Italy).

### 4.2 Checkerboard assay

The combinatorial effect of Esc(1-21)-1c and the antibiotics was determined by the checkerboard titration method by adding combinations of two compounds in a serial-two fold dilution to the wells of a 96-multiwell plate containing 1 × 10^6^ colony-forming units (CFU)/mL of *P. aeruginosa* PAO1 in a final volume of 100 µL of Mueller-Hinton (MH) broth. The plate was then incubated for about 16 h at 37°C. To calculate the Fractional Inhibitory Concentration Index (FICI) for the combination of two compounds the following formula was used:

$$\text{FICI}={\text{FIC}}_{A}+{\text{FIC}}_{B}=A\;/\;{\text{MIC}}_{A}+B\;/\;{\text{MIC}}_{B}$$

where A and B are the MICs of drug A and drug B in the combination, while MIC_A_ and MIC_B_ represent the MIC values of the compounds alone. The interpretation of the FICI was the following: FICI ≤0.5, synergy; 0.5<FICI ≤1, additivity, 1 < FICI ≤2, no interaction, FICI >2, antagonism (Pollini et al., 2017). The combination of Esc(1-21)-1c and tetracycline against the clinical isolate of *P. aeruginosa* was conducted as described above. All the experiments were performed in three independent replicates.

### 4.3 Differential proteomic analysis

*P. aeruginosa* PAO1 cells were grown in 3 mL of Luria Bertani (LB) medium until an optical density (OD, λ= 590 nm) of 0.5 was reached (corresponding to ∼1 × 10^8^ CFU/mL) and treated with 60 μg/mL (25 µM) of Esc (1-21)-1c at 37 °C for 3 h under stirring. Untreated cells were used as control and the experiment was performed in duplicate. Pellets were collected by centrifugation at 4°C for 10 min at 4,700 x g, resuspended in phosphate buffer saline (PBS), 5% sodium dodecyl sulfate (SDS) and 1 mg/mL lysozyme, and lysed by sonication. Samples were then centrifuged at 4 °C for 30 min at 14000 x g to collect unlysed cells and cell debris, while the recovered supernatant was quantified by Pierce™ BCA protein Assay Kit purchased from Thermo Scientific (Rockford-USA).

Fifty µg of each sample were digested with trypsin onto S-trapTM micro spin column, according to the manufacturer (Protifi, Huntington, NY). The obtained peptide mixtures were analyzed by LC-MS/MS using a LTQ Orbitrap XL coupled to a nanoLC system (ThermoFisher Scientific, Waltham, MA). All peptide mixtures were fractionated onto a C18 capillary reverse-phase column (200 mm length, 75 μm ID, 5 μm biosphere), using a non-linear 5%–50% gradient for eluent B (0.2% formic acid in 95% acetonitrile) in A (0.2% formic acid and 2% acetonitrile in MilliQ water) over 260 min.

MS/MS analyses were performed in Data-Dependent Acquisition (DDA) mode by fragmenting the 10 most intense ions in Collision-induced dissociation (CID) modality. All samples were run in duplicates. The obtained data were analyzed with MaxQuant (v.1.5.2.8) using UniProt *P. aeruginosa* as database for Andromeda search (Cox et al., 2011). Proteins were identified by selecting minimum 2 peptides, at least 1 unique, and considering methionine oxidation and pyroglutamate formation at the N-terminal glutamine as variable modifications. The accuracy for the first search was set at 10 ppm, then lowered to 5 ppm in the main search; 0.01 False Discovery Rate (FDR) was used, with a reverse database as decoy; retention time alignment and second peptide search functions were allowed. Fold changes (FCs) were calculated according to Label Free Quantification (LFQ) values.

Protein–protein interaction (PPI) networks were analyzed using the STRING program version 12.0 (string-db.org) which predicts PPI employing a mixture of prediction approaches and a combination of experimental data (textmining, experiments, databases, co-expression, neighborhood, gene fusion, co-occurrence). The mass spectrometry proteomics data have been deposited to the ProteomeXChange Consortium via the PRIDE (Perez-Rivero et al., 2022) partner repository with the dataset identifier PXD046086.

### 4.4 Gene expression analysis

RNA extraction has been performed as previously described (Letizia et al., 2022) on *P. aeruginosa* strains treated or not with Esc(1-21)-1c. For each condition, RNA was extracted from three independent bacterial cultures (biological triplicates). Strains were grown in 50 mL LB at 37 °C with shaking (200 rpm) until reaching an optical density (OD_590_) of 0.5. Subsequently, strains were incubated at 37°C with 60 μg/mL (25 µM) Esc(1-21)-1c dissolved in distilled sterile H_2_O or with an equal amount of distilled sterile H_2_O. RNA was extracted from bacterial cultures after 3 h of treatment. For each sample, 1 mL of culture was collected and incubated for 5 min at room temperature (RT) after the addition of 2 mL RNA Protect Bacteria Reagent (Qiagen). Bacterial suspensions were then centrifuged at 20°C for 20 min at 4,000 rpm, 20°C. The resulting pellets were suspended in 570 µL of TE buffer (10 mM Tris-HCl, 1 mM ethylenediaminetetraacetic acid; pH 8.0) and lysed by the addition of 1 mg/mL lysozyme and by sonication (2 cycles of 10 s pulsed sonication for each sample). RNA was extracted from lysed bacterial samples by using the RNeasy Minikit (Qiagen); on-column DNase I digestion step was also performed. DNA removal was obtained by treating eluted RNA for 1 h at 37 °C with 0.2 U/mg TURBO DNase (Ambion) and 0.4 U/mg SUPERase-In (Ambion). The RNeasy Column Purification kit (Qiagen) was used to purify RNA. The lack of DNA was verified by means of PCR analysis performed with the primer pair FW*PpqsB* and RV*PpqsB* (Supplementary Table S3). The NanoDrop 2000 spectrophotometer (Thermo Fisher Scientific) was used to quantify RNA.

One- µg of purified RNA was used to synthesize cDNA by using the iScript Reverse Transcription Supermix kit (Bio-Rad). The NanoDrop 2000 spectrophotometer (Thermo-Fisher Scientific) was used to quantify cDNA.

qRT-PCR analysis was performed on cDNA samples by using the iTaq Universal SYBR Green Supermix kit (Bio-Rad), and the AriaMX thermocycler (Agilent). To avoid amplification of nonspecific *P. aeruginosa* PAO1 cDNA, the primers employed in this analysis (Supplementary Table S3) were designed using Primer-BLAST (www.ncbi.nlm.nih.gov/tools/primer-blast). To normalize the qRT-PCR data and to determine the relative fold change (FC) in gene expression, the 16S rRNA was used as the internal control in every single run (Letizia et al., 2022) and the 2^−ΔΔCT^ method was employed. Mean FC values and standard deviations were calculated from three independent experiments.

### 4.5 Quantitative analysis by LC-MS/MS in MRM scan mode

Cell cultures were treated for 3 h in the presence of 64 μg/mL tetracycline or 64 μg/mL tetracycline plus 60 μg/mL Esc(1-21)-1c, using the untreated cells as control. The pellets were resuspended in water and subjected to three cycles of freezing and sonication. Protein precipitation occurred in cold methanol for 30 min at −20°C. Following centrifugation, the supernatants were recovered, dried in SpeedVac and used for the metabolomic study, while the pellets were used for the proteomic analysis.

The metabolomic samples were resuspended in acidified water and desalted using Oasis HLB cartridges. Metabolomic analyses were performed by LC-MS/MS by injecting 1 µL of supernatant and using the AB-sciex 5500 QTRAP^®^ system with a HPLC chromatography system Exion LC™. The mobile phase was generated by mixing eluent A (0.1% formic acid in water) and eluent B (0.1% formic acid in acetonitrile) with a flow rate equal to0.200 mL/min. Chromatographic gradient was 5% B for 1 min, then from 5% to 90% B in 4 min, and returned to 5% B in 2 min. Selected analytes were analysed using LC-MS/MS in MRM mode, and Turbo VTM ion source in positive ion mode. The Supplementary Table S4 provides a list of precursor ion, product ions, collision energy and declustering potential parameters. The mass chromatogram data obtained for the specific metabolite were analysed using Skyline software (20.2–64 bit version MacCoss Lab Software, University of Washington, United States).

The content of tetracycline was quantified using the calibration curve (See Supplementary Table S5; Supplementary Figure S2).

The protein pellets were solubilized in 6 M urea and 50 mM ammonium bicarbonate and subjected to protein hydrolysis by in-solution digestion with trypsin (Illiano et al., 2021). The peptide mixtures were desalted using custom-made chromatographic microcolumns. Skyline software was used to select the best precursor ion–product ion transitions, collision energy (CE), dwell time (minimal 5 ms) and cone voltage (35 V) (Di Somma et al., 2022). The selection of prototypic peptides for each protein target was calculated by matching data from the Skyline software (Abbatiello et al., 2013) and those collected in online repositories, e.g., SRM Atlas. The specific peptides were the following: MexB = FLMLAAQNPALQR; MexA = IITEGLQFVQPGVEVK; OprM = ADQAQLQLTK.

Peptide mixtures were analysed by LC-MS/MS in MRM positive ion mode using a Xevo TQ-S (Waters, Milfors, MA, United States) equipped with an IonKey UPLC Microflow Source coupled to an UPLC Acquity System (Waters, Milfors, MA, United States). One μL of the peptide mixtures was separated on a TS3 1.0 mm × 150 mm analytical RP column (Waters, Milford, MA, United States) at 45 °C at a flow rate of 3 μL/min using 0.1% formic acid in water (LC-MS grade) as eluent A and 0.1% formic acid in acetonitrile as eluent B. The peptide mixtures were separated by a linear gradient of eluent B in A from 7% to 95% in 55 min.

### 4.6 Statistical analysis

Quantitative data, collected from independent replicates, were expressed as the mean ± standard deviation (SD). Statistical analysis was calculated using PRISM 8.0.1 software (GraphPad, San Diego, CA) to perform *t*-test, one-way or two-way analysis of variance (ANOVA). Differences with *p* values < 0.05 were considered to be statistically significant and the levels of this significance were indicated in the legends to the figures.

## 5 Conclusion

In this paper, we have demonstrated a novel function of Esc(1-21)-1c and AMPs in general, that is the ability to significantly reduce MexAB-OprM efflux pump production in *P. aeruginosa* and increase the synthesis of OprD. This opens up possibilities for the development of alternative therapeutic strategies based on the combined use of AMPs/antibiotics to re-evaluate conventional drugs that are no longer considered highly effective for treating *pseudomonas* infections, by decreasing their efflux from cells, while enhancing their cellular uptake.

## Data Availability

The mass spectrometry proteomics data have been deposited to the ProteomeXChange Consortium via the PRIDE partner repository with the dataset identifier PXD046086.
