# Supplementary material for: The antimicrobial peptide Esc(1-21)-1c increases susceptibility of Pseudomonas aeruginosa to conventional antibiotics by decreasing the expression of the MexAB-OprM efflux pump
Source: Front Chem. 2023 Oct 24;11:1271153. doi: 10.3389/fchem.2023.1271153 (PMC10628714; doi:10.3389/fchem.2023.1271153)
Supplement: Supplementary file 1 [file DataSheet1.docx]

Supplementary Material

The antimicrobial peptide Esc(1-21)-1c increases susceptibility of *Pseudomonas aeruginosa* to conventional antibiotics by decreasing the expression of the MexAB-OprM efflux pump

**Carolina Canè^1¥^, Bruno Casciaro^2¥^, Angela Di Somma^1,3^, Maria Rosa Loffredo^2^, Elena Puglisi^2^, Gennaro Battaglia^1^, Marta Mellini^4^, Floriana Cappiello^2^, Giordano Rampioni^4,5^, Livia Leoni^4^, Angela Amoresano^1^, Angela Duilio^1*^, Maria Luisa Mangoni^2*^**

^1^Department of Chemical Sciences, University of Naples “Federico II”, Naples, Italy;
^2^Laboratory affiliated to Pasteur Italia-Fondazione Cenci Bolognetti, Department of Biochemical Sciences, Sapienza University of Rome, Italy;
^3^CEINGE Biotecnologie Avanzate, Naples, Italy;
^4^Department of Science, University “Roma Tre”, Rome, Italy
^5^IRCCS Fondazione Santa Lucia, Rome, Italy

^¥^These authors contributed equally to this work and share first authorship

***Correspondence to:** [angela.duilio@unina.it](mailto:angela.duilio@unina.it) (A.D.) and [marialuisa.mangoni@uniroma1.it](mailto:marialuisa.mangoni@uniroma1.it) (M.L.M.)

Supplementary Figure S1. Growth of *P. aeruginosa* PAO1 (measured as optical density) after 16 h treatment with a sub-inhibitory concentration of Esc(1-21)-1c (i.e., 25 µM) with respect to untreated control cells.

Equation 1: FICI index calculations for Esc(1-21)-1c and tobramycin combination

$$\frac{6.8}{6.8}+\frac{1}{1}=2$$

Equation 2: FICI index calculations for Esc(1-21)-1c and ceftazidime combination

$$\frac{3.4}{6.8}+\frac{1}{8}=0.625$$

Equation 3: FICI index calculation for Esc(1-21)-1c and erythromycin

$$\frac{1.7}{6.8}+\frac{32}{256}=0.375$$

Equation 4: FICI index calculation for Esc(1-21)-1c and chloramphenicol combination

$$\frac{0.85}{6.8}+\frac{4}{32}=0.25$$

Equation 5: FICI index calculation for Esc(1-21)-1c and tetracycline combination

$$\frac{1.7}{6.8}+\frac{1}{8}=0.375$$

Table S1. *P. aeruginosa* PAO1 up-regulated proteins

| SwissProt code | Protein name | Gene | FC |
| --- | --- | --- | --- |
| O54439 | Acyl carrier protein 1 | *acpP1* | 6,81 |
| Q9I3C5 | Chaperone protein HtpG | *htpG* | 5,1 |
| Q59638 | Dihydrolipoyllysine-residue acetyltransferase | *aceF* (*aceB*) | 3,3 |
| Q9HV55 | Translation initiation factor IF-2 | *infB* | 3,24 |
| Q51567 | Succinate--CoA ligase [ADP-forming] subunit alpha | *sucD* | 2,88 |
| Q02RW1 | Nucleoside diphosphate kinase | *ndk* | 2,73 |
| Q02T68 | 50S ribosomal protein L5 | *rplE* | 2,68 |
| Q9HVL6 | 50S ribosomal protein L21 | *rplU* | 2,64 |
| Q02UU0 | Alkyl hydroperoxide reductase C | *ahpC* | 2,63 |
| Q9HVA2 | Ketol-acid reductoisomerase (NADP(+)) | *ilvC* | 2,6 |
| Q9I3D1 | Dihydrolipoyl dehydrogenase | *lpdG* | 2,44 |
| Q9I0A2 | 50S ribosomal protein L20 | *rplT* | 2,28 |
| Q02T73 | 50S ribosomal protein L16 | *rplP* | 2,24 |
| Q59637 | Pyruvate dehydrogenase E1 component | *aceE* | 2,21 |
| Q02T88 | 50S ribosomal protein L7/L12 | *rplL* | 2,14 |
| B7UVD3 | Succinate--CoA ligase [ADP-forming] subunit beta | *sucC* | 2,09 |
| Q02NB5 | Isocitrate dehydrogenase [NADP], IDH | *icd* | 2,05 |
| Q02FR0 | Protein GrpE (HSP-70 cofactor) | *grpE* | 2,02 |
| P34750 | Fimbrial assembly protein PilQ | *pilQ* | 1,99 |
| P08308 | Ornithine carbamoyltransferase, catabolic | *arcB* | 1,95 |
| Q02PG5 | Glyceraldehyde-3-phosphate dehydrogenase | *gap2* | 1,94 |
| Q9HWD2 | Elongation factor G 1, EF-G 1 | *fusA* | 1,92 |
| Q9HT20 | ATP synthase subunit beta | *atpD* | 1,87 |
| Q9HWC9 | DNA-directed RNA polymerase subunit beta | *rpoC* | 1,86 |
| Q9I2V5 | Aconitate hydratase B, ACN, Aconitase | *acnB* | 1,76 |
| Q9HZP6 | Electron transfer flavoprotein subunit beta | *etfB* | 1,72 |
| P57668 | Thiol peroxidase, Tpx | *tpx* | 1,71 |
| O82851 | Elongation factor Ts, EF-Ts | *tsf* | 1,71 |
| Q9HW91 | Methyl-accepting chemotaxis protein PctB | *pctB* | 1,71 |
| Q02PH8 | Fatty acid oxidation complex subunit alpha | *fadB* | 1,67 |
| Q9HZE0 | NAD-specific glutamate dehydrogenase | *gdhB* | 1,68 |
| Q9I5Y1 | Fructose-bisphosphate aldolase | *fba (fda)* | 1,67 |
| P26480 | RNA polymerase sigma factor RpoD | *rpoD* | 1,66 |
| Q02DF2 | ATP synthase subunit alpha | *atpA* | 1,65 |
| P09591 | Elongation factor Tu, EF-Tu | *tufA; tufB* | 1,62 |
| Q9HT21 | ATP synthase epsilon chain | *atpC* | 1,59 |
| Q9HU15 | Beta-ketoacyl-[acyl-carrier-protein] synthase | *fabY* | 1,54 |
| Q02FT1 | 30S ribosomal protein S15 | *rpsO* | 1,53 |
| Q02T87 | DNA-directed RNA polymerase subunit beta, | *rpoB* | 1,52 |
| O54438 | 3-oxoacyl-[acyl-carrier-protein] reductase FabG | *fabG* | 1,47 |
| Q9HVI7 | Serine hydroxymethyltransferase 3 | *glyA2* | 1,46 |
| P31961 | Phosphogluconate dehydratase | *edd* | 1,45 |
| O82850 | 30S ribosomal protein S2 | *rpsB* | 1,45 |
| P32722 | Porin D | *oprD* | 1,45 |
| Q9HVC5 | Ribose-phosphate pyrophosphokinase, RPPK | *prs* | 1,43 |
| P04739 | Type IV major pilin protein PilA (Pilin) | *pilA* (*fimA*) | 1,35 |
| Q9HT16 | ATP synthase subunit b | *atpF* | 1,31 |
| Q02KU3 | Trigger factor, TF | *tig* | 1,3 |
| Q02H07 | 50S ribosomal protein L13 | *rplM* | 1,27 |
| Q02RA9 | CTP synthase | *pyrG* | 1,25 |
| Q02F86 | 50S ribosomal protein L9 | *rplI* | 1,25 |
| Q9HVN5 | Chaperone protein ClpB | *clpB* | 1,25 |
| Q02T90 | 50S ribosomal protein L1 | *rplA* | 1,25 |
| P13981 | Arginine deiminase, ADI | *arcA* | 1,24 |
| O52762 | Catalase | *katA* | 1,21 |
| P24474 | Nitrite reductase | *nirS* | 1,21 |
| Q02FT2 | Polyribonucleotide nucleotidyltransferase | *pnp* | 1,21 |

Table S2. *P. aeruginosa* PAO1 down-regulated proteins.

| SwissProt code | Protein name | Gene | FC |
| --- | --- | --- | --- |
| P37798 | Biotin carboxylase | *accC* (*fabG*) | 0,80 |
| Q9I407 | Glutaminase-asparaginase | *ansB* | 0,79 |
| Q02RL5 | 50S ribosomal protein L19 | *rplS* | 0,78 |
| P52477 | Multidrug resistance protein MexA | *mexA* | 0,78 |
| Q02T91 | 50S ribosomal protein L11 | *rplK* | 0,78 |
| O52759 | 30S ribosomal protein S4 | *rpsD* | 0,77 |
| Q02T66 | 30S ribosomal protein S8 | *rpsH* | 0,77 |
| Q02H08 | 30S ribosomal protein S9 | *rpsI* | 0,76 |
| Q9HVT2 | Alpha-2-macroglobulin homolog | *PA4489* | 0,76 |
| Q9HU56 | Protein-export protein SecB | *secB* | 0,72 |
| P38100 | Carbamoyl-phosphate synthase large chain | *carB* | 0,71 |
| Q02GB4 | 30S ribosomal protein S20 | *rpsT* | 0,71 |
| Q9I6G2 | UPF0339 protein PA0329 | *PA0329* | 0,71 |
| Q02E46 | 50S ribosomal protein L28 | *rpmB* | 0,71 |
| Q9HZA6 | Motility hub protein FimV | *fimV* | 0,70 |
| O52761 | 50S ribosomal protein L17 | *rplQ* | 0,64 |
| Q9HXY5 | Skp-like protein | *PA3647* | 0,63 |
| Q02V73 | Glycine--tRNA ligase beta subunit | *glyS* | 0,61 |
| Q9I5V8 | 30S ribosomal protein S21 | *rpsU* | 0,60 |
| Q02T67 | 30S ribosomal protein S14 | *rpsN* | 0,60 |
| Q02GB0 | 50S ribosomal protein L27 | *rpmA* | 0,60 |
| Q02T78 | 50S ribosomal protein L23 | *rplW* | 0,58 |
| Q9LCT3 | Protein translocase subunit SecA | *secA* | 0,56 |
| Q02RY8 | Probable cytosol aminopeptidase | *pepA* | 0,54 |
| Q02T85 | 30S ribosomal protein S12 | *rpsL* | 0,55 |
| Q9HWF8 | 30S ribosomal protein S11 | *rpsK* | 0,53 |
| Q51487 | Outer membrane protein OprM | *oprM* | 0,53 |
| Q02T77 | 50S ribosomal protein L2 | *rplB* | 0,52 |
| Q02T70 | 50S ribosomal protein L14 | *rplN* | 0,51 |
| Q9HWF3 | 50S ribosomal protein L30 | *rpmD* | 0,49 |
| Q9HWE4 | 30S ribosomal protein S17 | *rpsQ* | 0,49 |
| Q02EW8 | 50S ribosomal protein L31 | *rpmE* | 0,49 |
| Q02T71 | 30S ribosomal protein S17 | *rpsQ* | 0,49 |
| P47205 | UDP-3-O-acyl-N-acetylglucosamine deacetylase | *lpxC* (*envA*) | 0,47 |
| Q9HZN4 | 50S ribosomal protein L32 | *rpmF* | 0,47 |
| Q9HXP9 | 30S ribosomal protein S16 | *rpsP* | 0,45 |
| Q9HWF2 | 30S ribosomal protein S5 | *rpsE* | 0,45 |
| Q9I0K4 | Isocitrate lyase | *PA2634* | 0,45 |
| Q9HTN9 | 50S ribosomal protein L33 | *rpmG* | 0,45 |
| Q02T63 | 30S ribosomal protein S5 | *rpsE* | 0,43 |
| Q9I6Z1 | GDP-polyphosphate phosphotransferase | *ppk2* | 0,43 |
| Q9I4Z4 | Peptidoglycan-associated lipoprotein, PAL | *oprL* | 0,40 |
| P13794 | Outer membrane porin F | *oprF* | 0,40 |
| Q02T72 | 50S ribosomal protein L29 | *rpmC* | 0,38 |
| Q9HXN2 | Phosphoribosylformylglycinamidine synthase | *purL* | 0,38 |
| Q9HWF4 | 50S ribosomal protein L15 | *rplO* | 0,34 |
| P57112 | Soluble pyridine nucleotide transhydrogenase | *sthA* | 0,30 |
| P52002 | Multidrug resistance protein MexB | *mexB* | 0,29 |
| Q9HYT6 | RNA polymerase-associated protein RapA | *rapA* | 0,26 |
| P11221 | Major outer membrane lipoprotein | *oprI* | 0,22 |
| Q9HUN0 | 30S ribosomal protein S18 | *rpsR* | 0,17 |

**Table S3**. Oligonucleotides used in this study.

| Name | Sequence (5’-3’) |
| --- | --- |
| FW16S | GAGAGTTTGATCCTGGCTCAG |
| RV16S | CTACGGCTACCTTGTTACGA |
| FW*oprM* | TCAACCTGCCGATCTTCACC |
| RV*oprM* | GAGCTGGTAGTACTCGTCGC |
| FW*oprD* | AAGACCATGCTGAAGTGGGG |
| RV*oprD* | CCTGCGTAGGTGGCATAGAG |
| FW*oprI* | GCAGCCACTCCAAAGAAACC |
| RV*oprI* | TACTTGCGGCTGGCTTTTTC |
| FW*mexA* | CGAAGGTCTCCCTGAAGCTG |
| RV*mexA* | AGGATGGCCTTCTGCTTGAC |
| FW*mexB* | ACCTGAGCAAGTGGTACGTG |
| RV*mexB* | CTTGACGATCTCCTCGACCG |
| FW*secA* | TCAGCCTGGACGACAAGTTC |
| RV*secA* | TTGCCCTCGACCTCTTCAAC |
| FW*pilQ* | AGCATCATCGCCTATCAGCC |
| RV*pilQ* | TCCCGATATTGCCGTCCTTG |
| FW*PpqsB* | CCGCTCGAGCGACCAGGGCTATCGCA |
| RV*PpqsB* | CCGGAATTCCTTATGCATGAGCTTCTCC |

**Relative quantification of proteins from *P. aeruginosa*.**

Table S4. MRM method for tetracycline analysis

| Analytes | Rt (min) | Precursor (m/Z) | Product (m/Z) | DP (V) | EP (V) | CE 1/2 (V) | CXP 1/2 (V) | Dwell (msec) |
| --- | --- | --- | --- | --- | --- | --- | --- | --- |
| Tetracycline | 5.4 | 445.0 | 410.0 | 36 | 10 | 27 | 12 | 200 |
| Tetracycline | 5.4 | 445.0 | 427.0 | 36 | 10 | 19 | 18 | 200 |

* Rt = Retention time

* DP = Declustering Potential

* CE = Collision Energy

* CXP = Collision Cell Exit Potential

Table S5. Calibration curve

| **Tetracycline µg/L** |  |  | **Response** | | **Average** | **St Dev** | **rsd%** | **LOD** | **LOQ** |  |
| --- | --- | --- | --- | --- | --- | --- | --- | --- | --- | --- |
|  |  | **1** | **2** | **3** |  |  |  |  |  |  |
| 7,81 |  | 65885 | 64625 | 61256 | 63922 | 2393,3 | 3,7 | 4,1 | 12,6 |  |
| 15,62 |  | 135008 | 129911 | 125708 | 130209 | 4657,2 | 3,6 |  |  |  |
| 31,25 |  | 369766 | 374440 | 346498 | 363568 | 14966,6 | 4,1 |  |  |  |
| 62,5 |  | 824587 | 860854 | 810975 | 832139 | 25782,9 | 3,1 |  |  |  |
| 125 |  | 1835519 | 1801510 | 1898211,28 | 1845080 | 49054,5 | 2,7 |  |  |  |

***rsd%** = Relative standard deviation

***LOD** = Limit of detection

***LOQ** = Limit of quantification


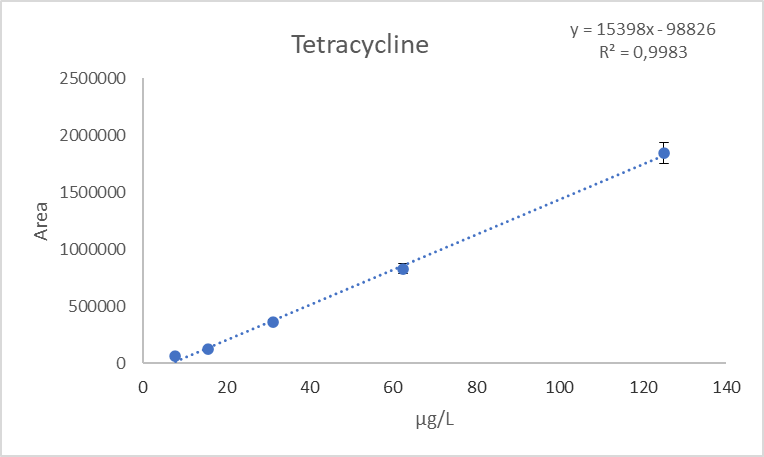


Supplementary Figure 2. The average of the calibration curve
